# Understanding the Ovarian Interrelationship with Low Antral Follicle Counts (AFC) in the In Vivo *Bos indicus* Cow Model: Unilateral and Bilateral Main AFC as Possible Biomarkers of Ovarian Response to Hormonal Synchronisation

**DOI:** 10.3390/biology11040523

**Published:** 2022-03-29

**Authors:** Warittha U-krit, Surasak Wadsungnoen, Punnawut Yama, Jakree Jitjumnong, Molarat Sangkate, Nalinthip Promsao, Napatsorn Montha, Paiwan Sudwan, Raktham Mektrirat, Julakorn Panatuk, Wilasinee Inyawilert, Payungsuk Intawicha, Pin-Chi Tang, Tossapol Moonmanee

**Affiliations:** 1 Department of Animal and Aquatic Sciences, Faculty of Agriculture, Chiang Mai University, Chiang Mai 50200, Thailand; u.warittha@gmail.com (W.U.-k.); punnawut_y@cmu.ac.th (P.Y.); pondforex39@gmail.com (M.S.); paejoojienlt@gmail.com (N.P.); napatsorn_mont@cmu.ac.th (N.M.); 2 Chiang Mai College of Agriculture and Technology, Sanpatong 50120, Thailand; 3 Phrae Cooperative Quality Beef Ltd., Nong Muang Kai 54170, Thailand; surasak.w@gmail.com; 4 Department of Animal Science, National Chung Hsing University, Taichung 40227, Taiwan; j.jakree105@gmail.com (J.J.); pctang@dragon.nchu.edu.tw (P.-C.T.); 5 Department of Anatomy, Faculty of Medicine, Chiang Mai University, Chiang Mai 50200, Thailand; paiwan.sudwan@cmu.ac.th; 6 Department of Veterinary Biosciences and Veterinary Public Health, Faculty of Veterinary Medicine, Chiang Mai University, Chiang Mai 50100, Thailand; raktham.m@cmu.ac.th; 7 Faculty of Animal Science and Technology, Maejo University, Chiang Mai 50290, Thailand; panatuk@gmail.com; 8 Department of Agricultural Science, Faculty of Agriculture, Natural Resources and Environment, Naresuan University, Phitsanulok 65000, Thailand; wilasineei@nu.ac.th; 9 Division of Animal Science, School of Agriculture and Natural Resources, University of Phayao, Phayao 56000, Thailand; payungsuk.in@up.ac.th; 10 The iEGG and Animal Biotechnology Center, National Chung Hsing University, Taichung 40227, Taiwan; 11 Innovative Agriculture Research Center, Faculty of Agriculture, Chiang Mai University, Chiang Mai 50200, Thailand

**Keywords:** antral follicle growth, dominant follicle, mono-ovulatory animal species, ovarian biology, transrectal ultrasonography

## Abstract

**Simple Summary:**

The number of growing antral follicles visualised by ultrasound generates an antral follicle count (AFC), which can predict the number of female gametes remaining in an animal’s ovary. However, important information on ovarian biology with regard to the ovarian interrelationship with the main number of antral follicle populations (mAFC) is scarce; therefore, it is important to uncover additional information on ovarian biology that can be helpful in predicting the ovarian response after hormonal stimulation in mono-ovulatory animal species. Thus, mono-ovulatory *Bos indicus* beef cows with low numbers of AFC were induced using a hormonal programme in an effort to increase the understanding of the interrelationship with unilateral mAFC (main number of antral follicle populations appearing on only one side of the ovary). Based on the odds ratio, our results emphasise that the likelihood of an ovarian response was higher in cows with unilateral mAFC relationships on the day of exogenous hormonal stimulation.

**Abstract:**

The antral follicle count (AFC) is a test in which the number of oocyte-containing follicles that are developing in both ovaries are visually counted. The count of these follicles strongly relates to the population of the growing follicle reserve on the ovaries. However, the importance of the main number of antral follicle populations (mAFC) in mono-ovulatory animal species has yet to be completely elucidated. Moreover, the investigation of the ovarian interrelationship with unilateral mAFC (main number of antral follicle populations appearing on only one side of the ovary) and bilateral mAFC (main number of antral follicle populations appearing in equivalent numbers on both sides of the ovary) and how understanding this interrelationship can offer possible indicators of ovarian response to hormonal induction have not yet been investigated in mono-ovulatory *Bos indicus* beef cows. The aim of this study is to investigate the different ovarian interrelationships of mAFC (unilateral and bilateral mAFC) at the time of exogenous hormonal stimulation on the total number of AFC (left and right ovaries) at the beginning of the hormonal protocol for ovarian stimulation and ovarian response at the completion of exogenous hormonal stimulation as well as their usefulness as possible biomarkers of successful hormonal stimulation in *Bos indicus* beef cattle. Beef cows (*n* = 104) with low total numbers of AFC (4.7 ± 2.4 follicles) were stimulated with a gonadotropin-releasing hormone-progesterone-prostaglandin F_2α_-based protocol. At the beginning of the hormonal protocol, ovarian ultrasound scans were performed to evaluate AFC from both ovaries of cows. Beef cows were divided into two groups, unilateral (*n* = 74) and bilateral mAFC (*n* = 30), according to the ovarian interrelationship. At the completion of the hormonal stimulation, ovarian ultrasound scans were performed to evaluate the dominant follicle (DF) and cows with DF > 8.5 mm in diameter emerging on their ovaries were defined as having experienced a response to hormonal stimuli. There was a difference of 19.1% between *Bos indicus* cows bearing unilateral mAFC that produced an increase in ovarian response (odds ratio = 2.717, *p* < 0.05) compared to the responsive rate of cows displaying bilateral mAFC (82.4% vs. 63.3%). In unilateral mAFC, cows bearing mAFC ipsilateral to the ovary of dominant follicle (DF) had a higher responsive rate than cows bearing mAFC contralateral to the DF ovary (50.0% vs. 32.4%, *p* < 0.05). In mAFC ipsilateral to the DF ovary, pregnancy rates were greatest in cows bearing mAFC and DF on the right ovary compared with cows bearing mAFC and DF on the left ovary (25.0% vs. 9.1%, *p* < 0.05). In primiparous and multiparous cows, unilateral mAFC occurs with a greater (*p* < 0.05) frequency than bilateral mAFC (69.0% and 72.0% vs. 31.0% and 28.0%, respectively). In unilateral mAFC, primiparous cows bearing mAFC ipsilateral to the DF ovary had a greater responsive rate than primiparous cows bearing mAFC contralateral to the DF ovary (55.0% vs. 20.0%, *p* < 0.05). In mAFC ipsilateral to the DF ovary, responsive and pregnancy rates were greatest (*p* < 0.05) in multiparous cows bearing mAFC and DF on the right ovary compared with multiparous cows bearing mAFC and DF on the left ovary (58.1% and 22.6% vs. 25.8% and 3.2%, respectively). Furthermore, there was a positive correlation between the mean diameter of AFC at the time of the exogenous hormonal trigger and the mean diameter of DF at the completion of hormonal synchronisation (*p* < 0.05). Our findings emphasise that the ovarian interrelationship with unilateral mAFC at the time of the hormonal trigger might be a promising biomarker for predicting success in ovarian response to hormonal stimulation of mono-ovulatory *Bos indicus* beef cows with low AFCs.

## 1. Introduction

Understanding reproductive biology in females is a crucial element of controlling reproductive functions in animals [1,2,3,4] and a greater body of knowledge about ovarian biology is essential to enhancing applied reproduction in female animals [5]. Female farm animals have provided important information about reproductive biology not only in rare and endangered animal species [6], but also in humans [7,8]. In the bovine model, intraovarian relationships between two ovarian structures (follicles and corpus luteum) have been observed in *Bos taurus* dairy heifers [9] and cows [10] and in *Bos indicus* beef cows [11], and the control of the ovarian development of the future dominant follicle (DF). In the *Bos indicus* model, the interaction of one ovary side (left or right) with the number of follicles per ovary was observed in beef cows [12]. Due to the importance of ovarian structures, measurements of follicular populations prior to stimulation have been applied to predict the ovarian response in cattle [13]. Under the evaluation of reproductive organs using invasive methods, the antral follicle count (AFC) has been used to reliably predict the number of morphologically healthy oocytes and follicles in both ovaries of young adult bovines [14]. To gain practical knowledge of ovarian biology by a noninvasive method, transrectal ultrasonography is considered to be an effective technique for the evaluation of the ovarian follicular structure in large female animals [15], and it can be used to generate important information regarding ovarian follicular populations, such as AFC. Nowadays, AFC, which represents the number of follicles visualised by ultrasonography, is widely used to predict the ovarian follicular reserve and fertility outcomes, not only in animal research [16,17], but also in medical research [18]. In an effort to apply assisted reproductive technology in improving fertility, AFC is an important indicator that can be used to predict ovarian response in women [19] and female cattle [20]. Investigations in dairy cows have identified associations between AFC and reproductive measures [21]. Moreover, high numbers of AFC are associated with high rates of in vitro embryo production in beef cows [22]. On the other hand, low numbers of AFC are negatively related to fertility in dairy cows [23]. On the basis of these observations, there are still answers to two questions to be added to the body of knowledge regarding ovarian biology. Specifically, with regard to the ovarian interrelationship when unilateral mAFC (main number of antral follicle populations appears on only one side of the ovary) and bilateral mAFC (main number of antral follicle populations appears in equivalent numbers on both sides of the ovary) emerge on ovaries of mono-ovulatory *Bos indicus* cows:

(1) What are the impacts of unilateral and bilateral mAFC at the time of hormonal stimulation on ovarian response and fertility?

(2) What are the possible applications of unilateral and bilateral mAFC as biomarkers of ovarian response to hormonal synchronisation?

Unfortunately, the impact of low numbers of AFC on the ovarian response and pregnancy outcome at the time of hormonal stimulation is not clear in *Bos indicus* beef cows. Moreover, the ovarian interrelationship of unilateral and bilateral mAFC and the possible indicators of ovarian response to hormonal induction have not yet been investigated in *Bos indicus* beef cows.

Collectively, these observations led us to hypothesise that: (1) different ovarian interrelationships of AFC (unilateral and bilateral mAFC) at the time of hormonal stimulation would result in an altered ovarian response (emergence of DF ≥ 8.5 mm in diameter) at the completion of hormonal synchronisation; (2) the unilateral or bilateral main AFC can act as possible biomarkers of successful hormonal induction in mono-ovulatory *Bos indicus* beef cows with low numbers of AFC. The main objective of the present research, therefore, is to investigate the different ovarian interrelationships of AFC (unilateral and bilateral mAFC) on the total number of AFC (left and right ovaries) at the beginning of the hormonal protocol for ovarian stimulation and ovarian response at the completion of the hormonal stimulation period, as well as the possible biomarkers of successful hormonal stimulation in *Bos indicus* beef cows with low numbers of AFC.

## 2. Materials and Methods

### 2.1. In Vivo Bovine Model

All animal experiments complied with the Ethical Principles and Guidelines for the Use of Animals of the National Research Council of Thailand and were approved by the Animal Care and Use Committee of Maejo University (approval number: MACUC033A/2563). Crossbred Brahman (*Bos indicus*) beef cows (*n* = 104) with age of 65.7 ± 17.6 months and body condition score (BCS) of 2.6 ± 0.4 points (a 5-point scale from 1 (thin) to 5 (fat) [24]) were used in the experiments. Beef cows were raised indoors in a free-stall barn and fed fresh Ruzi grass (*Brachiaria ruziziensis*) ad libitum, and supplemented with a commercial concentrate. Clean drinking water and mineralised salt bricks were provided ad libitum. Before the start of this experiment, all cows were confirmed to have no clinically evident conditions of cystic ovary, dystocia, retained placenta, or metritis.

### 2.2. Determination of Ovarian AFC and Animal Group

**Figure 1:**
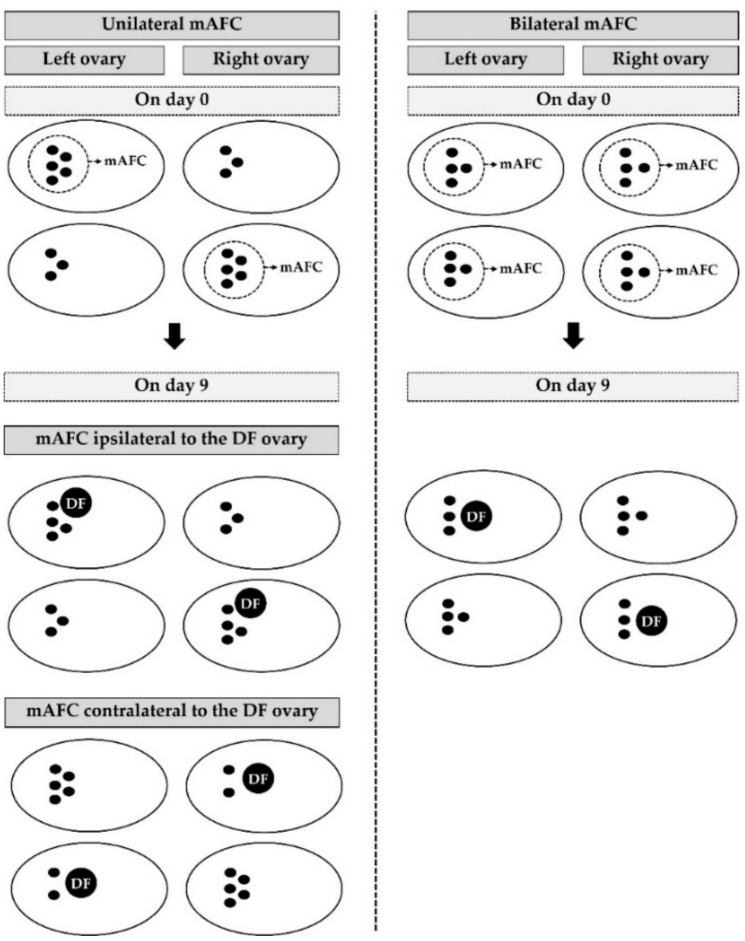
Illustration of the ovarian interrelationships between mAFC on the day of initiation of hormonal stimulation (day 0) and DF at the completion of hormonal stimulation (day 9) in *Bos indicus* cows (*n* = 104). Unilateral mAFC means that the number of antral follicle populations appears on only one side of the ovary. Bilateral mAFC means that the main number of antral follicle populations appears in equivalent numbers on both sides of the ovary. Black circles represent antral follicles (≥3.0 mm in diameter). AFC, antral follicle count; mAFC, main number of antral follicle populations; DF, dominant follicle.

At the beginning of the study, ovarian ultrasound scans (a 7.5 MHz linear-array ultrasonic probe; HS-1600V, Honda Electronics, Japan) were performed by a single operator to evaluate the ovaries (left and right sides) of each cow, and the relative position and dimensions of all follicles ≥ 3.0 mm in diameter (antral follicles) were sketched on ovarian charts. The sonogram of beef cows bearing antral follicles on their ovary is provided in the Supplementary Materials (Figure S1A). All antral follicles were counted from the ovaries (left and right sides) of each cow and selected *Bos indicus* beef cows with mean number of AFC of 4.7 ± 2.4 follicles (*n* = 104; 29 primiparous and 75 multiparous cows) were classified as having low numbers of AFC (≤15 follicles [25]). Thus, selected *Bos indicus* beef cows with low numbers of AFC were divided into two groups, unilateral (*n* = 74) and bilateral mAFC (*n* = 30), according to the ovarian interrelationship (Figure 1). Unilateral mAFC means that the main number of antral follicle populations appears on only one side of the ovary, while bilateral mAFC means that main number of antral follicle populations appears in equivalent numbers on both sides of the ovary (Figure 1). Furthermore, in unilateral mAFC, the relationship between mAFC at the beginning of the hormonal stimulation (day 0) and the specific side of the DF ovary at the completion of the hormonal stimulation (day 9) was used to subclassify a total of 74 beef cows into two groups: mAFC ipsilateral to the DF ovary (*n* = 44) and mAFC contralateral to the DF ovary (*n* = 30). The mAFC ipsilateral to the DF ovary means that the main number of antral follicle populations on day 0 appears on the same side of the DF ovary on day 9 (Figure 1). The mAFC contralateral to the DF ovary means that the main number of antral follicle populations on day 0 appears on the opposite side of the DF ovary on day 9 (Figure 1). In bilateral mAFC, relationships between location of ovarian structures (mAFC and DF) and side of the ovary were used to subclassify a total of 30 beef cows into two groups (Figure 1): mAFC and DF appearing on the left ovary (*n* = 15) and mAFC and DF appearing on the right ovary (*n* = 15). The mAFC and DF appearing on the left ovary means that the main number of antral follicle populations on day 0 and DF on day 9 appear on the left ovary (Figure 1). The mAFC and DF appearing on the right ovary means that the main number of antral follicle populations on day 0 and DF on day 9 appear on the right ovary (Figure 1). The ovarian interrelationship between mAFC at the beginning of the hormonal stimulation (day 0) and DF at the completion of the hormonal stimulation (day 9) in *Bos indicus* cows is illustrated in Figure 1.

### 2.3. Hormonal Protocol for Ovarian Stimulation and Determination of Ovarian Response

**Figure 2:**
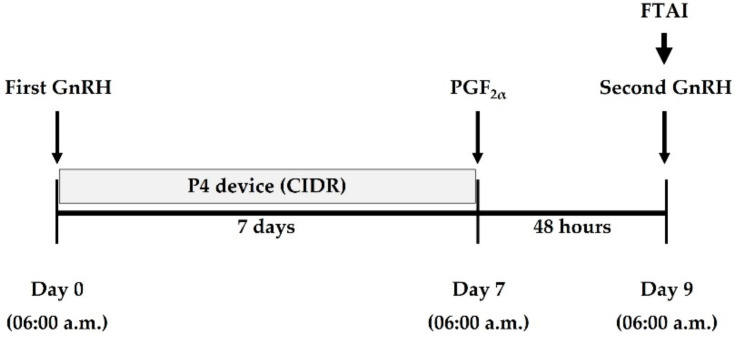
Schematic representation of hormonal protocol for ovarian stimulation. CIDR, controlled internal drug release; FTAI, fixed-time artificial insemination; GnRH, gonadotropin-releasing hormone; P4, progesterone; PGF_2α_, prostaglandin F_2α_.

At the beginning of the hormonal protocol for ovarian stimulation (day 0), a total of 104 selected *Bos indicus* beef cows with low numbers of AFC was implanted with an exogenous progesterone (P4)-releasing device with 1.38 g of P4 (controlled internal drug release (CIDR), Eazi-Breed; Zoetis Inc., Auckland, New Zealand), concurrent with an injection of a 10 µg dose (buserelin) of first gonadotropin-releasing hormone (GnRH, Receptal, MSD Animal Health, Wellington, New Zealand), and CIDR inserts were removed from the vagina on day 7 (Figure 2). On day 7, all beef cows received a 250 µg dose (cloprostenol) of prostaglandin F_2α_ (PGF_2α_, Estrumate, MSD Animal Health, New Zealand; Figure 2). On day 9, all beef cows received a 10 µg dose (buserelin) of second GnRH, concurrent with the fixed-time artificial insemination (FTAI) using a single dose of frozen-thawed semen (Figure 2). The completion of the hormonal synchronisation period was designated as day 9 (Figure 2).

At the completion of the hormonal stimulation period (day 9), ovarian ultrasound scans were performed by a single operator to evaluate the structures of the largest ovarian follicle (DF), and the relative position and dimensions of DF were sketched on ovarian charts. The sonogram of beef cows bearing DF on their ovary is provided in the Supplementary Materials (Figure S1B). On day 9, *Bos indicus* beef cows with DF > 8.5 mm in diameter emerging on their ovaries were defined as having experienced a response to hormonal stimuli, indicating successful hormonal stimulation [26,27]. The responsive rate was defined as the proportion of beef cows in which DF (>8.5 mm) emerged on their ovaries, divided by the total number of experimental beef cows.

### 2.4. Pregnancy Diagnosis

Beef cows were diagnosed as pregnant using transrectal ultrasonography on day 32 post-FTAI. Uterine ultrasound scans were performed by one examiner to determine the structures of the uterine horns. Beef cows were defined as pregnant when the embryonic vesicle could be found in the uterine horn. The sonograms of beef cows that were defined as pregnant and non-pregnant statuses at 32 days post-FTAI are provided in the Supplementary Materials (Figure S2). The pregnancy rate was defined as the proportion of beef cows that were diagnosed as pregnant on day 32 post-FTAI, divided by the total number of bred beef cows at FTAI.

### 2.5. Analysis of Data

The animal groups (unilateral and bilateral mAFC) were included in the statistical model as class variables, while age and BCS were also included in the statistical model as covariates. The Student’s *t* test was used to compare the means of the total number and diameter of antral follicles, and diameter of DF between beef cows with unilateral and bilateral mAFC, between mAFC ipsilateral and contralateral to the DF ovary, and between mAFC and DF appearing on the left and right ovaries. Data regarding number and diameter of antral follicles and diameter of DF are expressed as the mean ± standard error of the mean (SEM). Chi-square analysis was used to compare the frequency relationship and the percentages of the beef cows with DF > 8.5 mm in diameter emerging, as well as the pregnancy rate between beef cows with unilateral and bilateral mAFC, between mAFC ipsilateral and contralateral to the DF ovary, and between mAFC and DF appearing on the left and right ovaries. Linear regression was applied to evaluate the linear relationship between number of antral follicles and DF diameter, and between diameter of antral follicles and DF diameter. The odds ratio (OR) and 95% confidence intervals (CI) were used to evaluate the association between ovarian response and ovarian interrelationship, parity, BCS, and age. A probability of *p*-value ≤ 0.05 indicated a significant difference.

## 3. Results

### 3.1. Factors Relating to a Positive Ovarian Response in Bos indicus Cow Model

Based on the OR, the likelihood of an ovarian response was higher in *Bos indicus* cows with unilateral mAFC relationships on the day of the exogenous hormonal trigger (day 0). Compared to *Bos indicus* cows with bilateral mAFC relationships, cows bearing unilateral mAFC on their ovaries were more likely (82.4% vs. 63.3%) to experience a response (OR = 2.717; 95% CI = 1.061–6.953; *p* < 0.05; Table 1).

### 3.2. The Effect of Ovarian Interrelationship on AFC, DF, and Fertility in All Cows

#### 3.2.1. The Impact of Unilateral and Bilateral mAFC on Number and Diameter of AFC, Ovarian Response, and Pregnancy Outcome

Unilateral mAFC occurred with greater (*p* < 0.05) frequency than bilateral mAFC in *Bos indicus* cows (Table 2). On the day of the exogenous hormonal trigger (day 0), there were no differences in the total number and diameter of AFC on both ovaries between cows exhibiting unilateral and bilateral mAFC (Table 2). At the completion of the hormonal synchronisation period (day 9), there was no difference in the mean diameter of DF between unilateral and bilateral mAFC cows (Table 2). Interestingly, the percentage of cows with DF > 8.5 mm in diameter emerging at the completion of the hormonal stimulation period was higher (*p* < 0.05) in unilateral mAFC relationships than in bilateral mAFC relationships (Table 2). However, unilateral and bilateral mAFC relationships did not alter pregnancy rates on day 32 post-FTAI in cows subjected to the hormonal stimulation (Table 2).

#### 3.2.2. The Impact of the Specific Side of the mAFC and the DF Ovaries on Antral Follicle Parameters, Ovarian Response, and Pregnancy Rates in Unilateral mAFC Cows

In unilateral mAFC relationships, there were no differences in the mean number and diameter of AFC, mean diameter of DF, and pregnancy rates between cows bearing mAFC ipsilateral and contralateral to the DF ovaries (Table 3). Interestingly, the frequency relationship and percentage of cows with DF > 8.5 mm in diameter emerging were greater (*p* < 0.05) in *Bos indicus* cows bearing mAFC ipsilateral to the DF ovary than *Bos indicus* cows bearing mAFC contralateral to the DF ovary (Table 3).

In mAFC ipsilateral to the DF ovary, mAFC occurred with greater (*p* < 0.05) frequency in the right ovary than the left ovary of *Bos indicus* cows (Table 3). Additionally, both the percentage of cows with DF > 8.5 mm in diameter emerging and pregnancy rates were higher (*p* < 0.05) in *Bos indicus* cows bearing mAFC and DF on the right ovary than in cows bearing mAFC and DF on the left ovary (Table 3).

In mAFC contralateral to the DF ovary, there were no differences in all parameters of AFC and DF or in pregnancy rate (Table 3).

#### 3.2.3. The Impact of Location of Ovarian Structures and Side of the Ovary on Number and Diameter of AFC, Ovarian Response, and Pregnancy Rates in Bilateral mAFC Cows

In bilateral mAFC relationships, the frequency of mAFC and DF on the cow ovaries did not differ between the left ovary and the right ovary (Table 4). The location of mAFC and DF on the cow ovaries did not impact on any parameters of AFC and DF or on the pregnancy rate (Table 4).

### 3.3. The Effect of Ovarian Interrelationship on AFC, DF, and Fertility in Primiparous and Multiparous Cows

#### 3.3.1. Primiparous Cows

Unilateral mAFC occurred with greater (*p* < 0.05) frequency than bilateral mAFC in primiparous cows (Table 5). However, different ovarian interrelationships did not affect the parameters of AFC and DF or on the pregnancy rate in primiparous cows (Table 5).

In unilateral mAFC relationships, the location of mAFC and DF on the primiparous cow ovaries did not impact on any parameters of AFC and DF or on the pregnancy rate (Table 6). However, the percentage of cows with DF > 8.5 mm in diameter emerging was greater (*p* < 0.05) in primiparous cows bearing mAFC ipsilateral to the DF ovary than primiparous cows bearing mAFC contralateral to the DF ovary (Table 6).

In bilateral mAFC relationships, the frequency of mAFC and DF on the ovaries was higher (*p* < 0.05) in primiparous cows bearing mAFC and DF on the left ovary than in cows bearing mAFC and DF on the right ovary (Table 7). However, the total number of AFC was greater (*p* < 0.05) in primiparous cows bearing mAFC and DF on the right ovary than in cows bearing mAFC and DF on the left ovary (Table 7).

#### 3.3.2. Multiparous Cows

Unilateral mAFC occurred with greater (*p* < 0.05) frequency than bilateral mAFC in multiparous cows (Table 8). Nevertheless, unilateral and bilateral mAFC relationships did not alter parameters of AFC and DF or on pregnancy rate in multiparous cows (Table 8).

In unilateral mAFC relationships, there were no differences in all parameters of AFC and DF, in the percentage of cows with DF > 8.5 mm in diameter emerging or in the pregnancy rate between multiparous cows bearing mAFC ipsilateral and contralateral to the DF ovaries (Table 9). Interestingly, in mAFC ipsilateral to the DF ovary, mAFC occurred with greater (*p* < 0.05) frequency in the right ovary than the left ovary of multiparous cows (Table 9). Additionally, both the percentage of cows with DF > 8.5 mm in diameter emerging and pregnancy rates were higher (*p* < 0.05) in multiparous cows bearing mAFC and DF on the right ovary than in cows bearing mAFC and DF on the left ovary (Table 9).

In bilateral mAFC relationships, the frequency of mAFC and DF on the multiparous cow ovaries did not differ between the left ovary and the right ovary (Table 10). The location of mAFC and DF on the multiparous cow ovaries did not affect the total number of AFC, diameter of DF, and pregnancy rate (Table 10). However, the diameter of AFC was greater (*p* < 0.05) in multiparous cows bearing mAFC and DF on the right ovary than in cows bearing mAFC and DF on the left ovary (Table 10).

### 3.4. Association between AFC on the Day of Initiation of Hormonal Stimulation (Day 0) and DF on the Day of the FTAI (Day 9)

**Figure 3:**
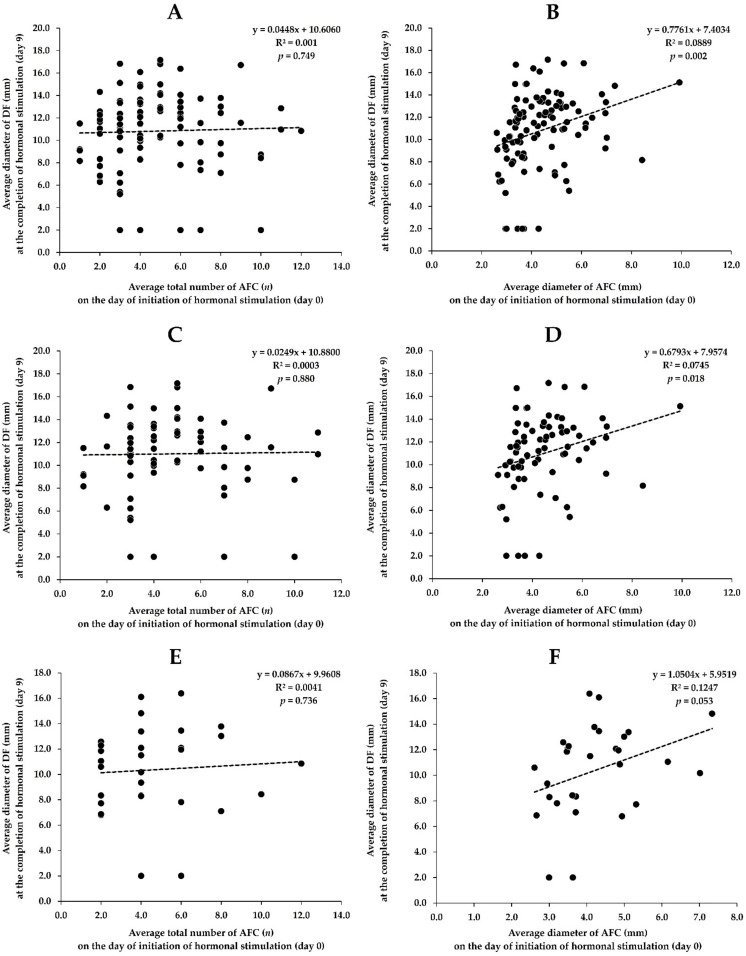
Associations between mean number and diameter of AFC on the day of initiation of hormonal stimulation (day 0) and mean diameter of DF at the completion of hormonal stimulation (day 9) in all *Bos indicus* cows ((**A**,**B**), *n* = 104) and in *Bos indicus* cows with unilateral ((**C**,**D**), *n* = 74) and bilateral mAFC ((**E**,**F**), *n* = 30) relationships.

Overall, there was no correlation between the mean number of AFC on the day of the initiation of hormonal stimulation (day 0) and the mean diameter of DF at the completion of the hormonal stimulation (day 9) in all *Bos indicus* cows (Figure 3A). Interestingly, there was a positive correlation (r = 0.301, *p* < 0.05) between the mean diameter of AFC on day 0 and the mean diameter of DF on day 9 (Figure 3B).

In *Bos indicus* cows with unilateral mAFC relationships, there was no association between the mean number of AFC on day 0 and the mean diameter of DF on day 9 (Figure 3C). Interestingly, there was a positive association (r = 0.275, *p* < 0.05) between the mean diameter of AFC on day 0 and the mean diameter of DF on day 9 (Figure 3D).

In *Bos indicus* cows with bilateral mAFC relationships, there were no relationships between the mean number of AFC on day 0 and the mean diameter of DF on day 9 (Figure 3E) and between the mean diameter of AFC on day 0 and the mean diameter of DF on day 9 (Figure 3F).

## 4. Discussion

There were three key findings in the present study of the in vivo *Bos indicus* cow model. First, *Bos indicus* cows bearing unilateral mAFC on their ovary at the initiation of hormonal stimulation were observed to have a greater ovarian response at the completion of the hormonal synchronisation period than that of cows bearing bilateral mAFC. Second, the ovarian response and pregnancy rates were greater in unilateral mAFC cows bearing mAFC ipsilateral to the DF ovary, and mAFC ipsilateral to the DF ovary was more frequent in the right ovary. Third, a linear correlation was found between the mean diameter of AFC on the day of the initiation of hormonal stimulation and the mean diameter of DF at the completion of the hormonal stimulation in mono-ovulatory *Bos indicus* cows. To the best of the researchers’ knowledge, this was the first investigation to provide data on ovarian interrelationship of unilateral mAFC as a possible biomarker of ovarian response to hormonal stimulation in the in vivo *Bos indicus* cow model.

The purpose of this study was to gain a better understanding of the ovarian interrelationships of unilateral mAFC in mono-ovulatory *Bos indicus* cows, especially in relation to the increased follicular response and, subsequently, DF emergence at the completion of the hormonal stimulation, not only through the hormonal stimulation itself, but also through the greater main population of antral follicles on only one side of the ovary (unilateral mAFC). Interestingly, regardless of whether the *Bos indicus* cattle were primiparous or multiparous, our findings strongly confirmed that a greater ovarian response was exhibited in cows bearing the main population of antral follicles on the same side of the ovary as the future DF appearance (mAFC ipsilateral to the DF ovary). Moreover, primiparous cows that presented with mAFC ipsilateral to the DF ovary had a greater ovarian response than primiparous cows bearing mAFC contralateral to the DF ovary. Under the dynamic reserve of antral growing follicles, previous studies have pointed out that the ovary side with a high reserve of primordial follicles displays a high AFC [28] and subsequent developmental potential to future DF [29]. Whereas past researchers have found that a positive association exists between the location relative to the antral follicle (6 mm) and future DF [29], the present study showed that antral follicles of mAFC on only one side of the ovary (unilateral mAFC) were available to produce a DF in the final stage of follicular development. This ovarian interrelationship was probably due to the fact that an increase in the number of antral follicles in the ovary of mono-ovulatory cattle may enhance the opportunity that one of the antral follicles could become the DF [29]. In primiparous cattle, a previous study reported a high repeatability of AFC for the same individuals as heifers and primiparous cows [30]. Moreover, the populations of antral follicles 3–7 mm in diameter were targets of the stimulatory treatment, and primiparous cows with a high density of antral follicles 3–7 mm in diameter at the initiation of the hormonal stimulation had high ovarian responses to the exogenous hormonal treatment [31,32]. This implies that the unilateral mAFC, indicated as the main ovarian reserve in cattle, reflected their responsive potential to hormonal stimulation. Thus, based on a high frequency relationship and ovarian response in unilateral mAFC, it is possible to infer that cows bearing mAFC on only one side of the ovary have a greater follicular activity due to a high density of antral growing follicles [33]. The ovarian microenvironment is crucial for antral follicle growth, such as vascularisation [34]. In fact, the supply of nutrients, hormones, and growth factors to follicular cells for supporting ovarian activity depends on an adequate blood flow [35]. As stated above, although ovarian vascularity was not evaluated in the present experiment, our findings supported the results of previous researchers who indicated that the growing follicle count correlated with the vascularised volume in the ovaries [36], and the relationship between ovarian vascularisation and the mitotic activity of follicular cells was observed predominantly in growing antral follicles of mono-ovulatory species [37,38,39]. Moreover, the findings of the present study represented the first direct demonstration of an intraovarian relationship between mAFC and DF in unilateral mAFC, in which the frequency (59.5% vs. 40.5%) and ovarian response (50.0% vs. 32.4%) were greater in cows bearing mAFC ipsilateral to the DF ovary than cows bearing mAFC contralateral to the DF ovary. This was possibly due to the close relationship between the development of AFC and future DF in mono-ovulatory species. As stated above, this idea was further supported by the finding that the number of smaller antral follicles 2–5 mm in diameter was greatly correlated with the number of larger antral follicles 2–10 mm in diameter [40] and only a few follicles of 6–10 mm in diameter being found in the early follicular phase [41]. Additionally, regardless of primiparous and multiparous *Bos indicus* cattle, our findings highlighted that mAFC ipsilateral to the DF ovary occurred with greater frequency in the right ovary than the left ovary (70.5% vs. 29.5%). In the preset study, the ovarian response and fertility were higher in multiparous cows bearing mAFC and DF on the right ovary than in multiparous cows bearing mAFC and DF on the left ovary (58.1% vs. 25.8%). This pattern of results was consistent with the previous reports that the ovulation of DF occurs with a higher frequency in the right ovary than the left ovary of mono-ovulatory species such as cows [42] and women [43]. Investigations in primiparous and multiparous dairy cows receiving the hormonal treatment for FTAI [10] have observed that the frequency of DF in the right ovary was greater than that in the left ovary at the completion of the hormonal stimulation (61.2% vs. 38.8%). Moreover, in multiparous beef cattle, Quail et al. [44] found that the total AFCs in multiparous beef cows are consistent within cows. The consistent difference in frequency is likely explained by a difference external to the ovary, such as the spatial proximity to other organs, angioarchitecture, or innervation that results in the biological activity of the right ovary as indicated by an increased volume of follicular fluid for antral follicles 4–13 mm, but not for antral follicles 1–3 mm [45]. Investigations in mono-ovulatory mares have observed that the mean number of antral follicle density was higher in the right ovary [46]. It is plausible that the right ovary has a greater volume and more AFC compared to the left ovary [47]. Although no evaluations of ovulation and oocyte quality in *Bos indicus* cows after hormonal stimulation were attempted in the present experiment, our pregnancy rate results from the in vivo cow study supported the findings of several researchers who reported that right-sided ovulation is more frequent than left-sided ovulation [48] and fertility from oocytes derived from the right ovary may be superior to oocytes from the left ovary [43] in mono-ovulatory species. Considering the former scenario, greater right-sided activity may explain a higher rate of ovarian response and fertility of *Bos indicus* cows with mAFC ipsilateral to the DF ovary. A similar ovarian interrelationship has been observed in the ovarian response after hormonal stimulation, in which the right ovarian response was superior to the left ovarian response in follicle recruitment, oocyte quality [49], and embryo production, with a subsequent increase in pregnancy outcomes [43]. In the *Bos taurus* cow model, more right-sided ovulation is not only a reason for this nonequivalent ovarian activity, but also for the greater development potential of right-sided oocytes [42]. Presumably, although the fundamental endocrine control is the same for the left and right ovaries, there are separate interovarian and intraovarian physiologic differences controlling the ovarian follicular development, locational side of ovulation, number and quality of oocytes, and fertility [50].

It is interesting to note that, across the ovarian interrelationship with unilateral and bilateral mAFC, a significant positive correlation was found between the mean diameter of AFC on the day of the exogenous hormonal trigger and the mean diameter of DF at the completion of the hormonal stimulation in mono-ovulatory *Bos indicus* cows. Subjectively, the most compelling explanation for the present set of findings is that *Bos indicus* cows with large-diameter AFC subjected to hormonal stimulation resulted in an increased diameter of DF and preovulatory follicle [51]. This strongly implies that there was a progressive increase in the follicular diameter of the antral follicle pool after stimulation with exogenous hormones. In agreement with the results of this study, the growth of antral follicles resulting in increased antral follicle diameters and diameters of DF correlated well with the size of the antral follicle pool (AFC) in mono-ovulatory species [41]. In fact, during hormonal synchronisation, most of the antral follicle reserves are required to grow in a coordinated fashion in response to exogenous gonadotropins to simultaneously accomplish functional and morphologic maturation in mono-ovulatory species such as cows [52] and women [53,54].

## 5. Conclusions

The present research contributes to a growing body of evidence suggesting that a unilateral mAFC relationship on the day of the initiation of hormonal stimulation might be a promising biomarker for estimating the functional ovarian reserve and predicting the response to exogenous hormones in the in vivo *Bos indicus* cow model with low AFC, because a positive association exists between mAFC and future DF. Moreover, a unilateral mAFC relationship might be indicative of predicting a response to exogenous hormones in both primiparous and multiparous *Bos indicus* cattle. However, much work remains to be performed before a full understanding of the extent of the unilateral mAFC relationship is established.

## Figures and Tables

**Table 1:** The OR for the risk factors contributing to ovarian response of in vivo *Bos indicus* cow model subjected to the hormonal stimulation (*n* = 104).

Variable	Responsive Cows (*n*)	Nonresponsive Cows (*n*)	Responsive Rate (%, *n*/*n*)	OR	95% CI	*p*-Value
Ovarian interrelationship (Ref = Bilateral mAFC)						
Bilateral mAFC ^1^	19	11	63.3 (19/30)			
Unilateral mAFC ^2^	61	13	82.4 (61/74)	2.717	1.061–6.953	0.037
Parity (Ref = Primiparous)						
Primiparous	20	9	69.0 (20/29)			
Multiparous	60	15	80.0 (60/75)	1.800	0.685–4.732	0.233
BCS (Ref = <2.5)						
<2.5	10	5	66.7 (10/15)			
2.5–3.0	62	18	77.5 (62/80)	1.722	0.523–5.671	0.371
>3.0	8	1	94.7 (8/9)	4.000	0.409–39.120	0.233
Age (Ref = <72 months)						
<72 months	41	16	71.9 (41/57)			
72–96 months	35	7	83.3 (35/42)	1.951	0.724–5.260	0.186
>96 months	4	1	80.0 (4/5)	1.561	0.161–15.089	0.700

^1^ Bilateral mAFC means that the main number of antral follicle populations appears in equivalent numbers on both sides of the ovary. ^2^ Unilateral mAFC means that the main number of antral follicle populations appears on only one side of the ovary. mAFC, main number of antral follicle populations; BCS, body condition score; OR, odds ratio; CI, confidence interval.

**Table 2:** Frequency of ovarian interrelationships and the impacts of ovarian interrelationships (unilateral and bilateral mAFC) on mean number and diameter of AFC (mean ± SEM), ovarian response, and pregnancy outcomes in in vivo *Bos indicus* cow model submitted to hormonal stimulation.

Items	Ovarian Interrelationships (*n* = 104)		*p*-Value
	Unilateral mAFC ^1^	Bilateral mAFC ^2^	
Total beef cows (*n*)	74	30	-
On day 0 ^3^			
Frequency relationship (%, *n*/*n*)	71.2 (74/104)	28.8 (30/104)	0.001
Mean number of AFC from left to right ovaries (*n*)	4.6 ± 0.27	4.7 ± 0.47	0.974
Mean diameter of AFC from left to right ovaries (mm)	4.5 ± 0.16	4.2 ± 0.21	0.303
On day 9 ^4^			
Beef cows DF > 8.5 mm in diameter (%, *n*/*n*)	82.4 (61/74)	63.3 (19/30)	0.037
Mean diameter of DF (mm)	11.0 ± 0.40	10.4 ± 0.63	0.412
On day 32 ^5^			
Pregnancy rate (%, *n*/*n*)	35.1 (26/74)	30.0 (9/30)	0.617

^1^ Unilateral mAFC means that the main number of antral follicle populations appears on only one side of the ovary. ^2^ Bilateral mAFC means that the main number of antral follicle populations appears in equivalent numbers on both sides of the ovary. ^3^ The day of initiation of hormonal stimulation. ^4^ The day of the FTAI (at the completion of hormonal stimulation). ^5^ The day of the post-FTAI. AFC, antral follicle count; mAFC, main number of antral follicle populations; FTAI, fixed-time artificial insemination.

**Table 3:** Frequency of unilateral mAFC relationships (mAFC ipsilateral and contralateral to the DF ovary) and impact on mean number and diameter of AFC (mean ± SEM), ovarian response, and pregnancy outcomes in in vivo *Bos indicus* cow model submitted to the hormonal stimulation.

Items	Unilateral mAFC (*n* = 74) ^1^					
	mAFC Ipsilateral to the DF Ovary			mAFC Contralateral to the DF Ovary		
	mAFC on the Left Ovary	mAFC on the Right Ovary	Total	mAFC on the Left Ovary	mAFC on the Right Ovary	Total
Total beef cows (*n*)	13	31	44	14	16	30
On day 0 ^2^						
Frequency relationship (%, *n*/*n*)	29.5 (13/44) ^b^	70.5 (31/44) ^a^	59.5 (44/74) ^A^	46.7 (14/30)	53.3 (16/30)	40.5 (30/74) ^B^
Mean number of AFC from left to right ovaries (*n*)	4.6 ± 0.60	4.7 ± 0.42	4.7 ± 0.35	4.6 ± 0.45	4.9 ± 0.70	4.6 ± 0.43
Mean diameter of AFC from left to right ovaries (mm)	4.3 ± 0.29	4.3 ± 0.26	4.3 ± 0.20	4.4 ± 0.21	5.0 ± 0.42	4.7 ± 0.25
On day 9 ^3^						
Beef cows DF > 8.5 mm in diameter (%, *n*/*n*)	27.3 (12/44) ^b^	56.8 (25/44) ^a^	50.0 (37/74) ^A^	36.7 (11/30)	43.3 (13/30)	32.4 (24/74) ^B^
Mean diameter of DF (mm)	11.1 ± 0.62	11.0 ± 0.63	11.0 ± 0.48	11.2 ± 1.03	10.8 ± 0.89	10.9 ± 0.68
On day 32 ^4^						
Pregnancy rate (%, *n*/*n*)	9.1 (4/44) ^b^	25.0 (11/44) ^a^	20.3 (15/74)	13.3 (4/30)	23.3 (7/30)	14.9 (11/74)

^1^ Unilateral mAFC means that the main number of antral follicle populations appears on only one side of the ovary. ^2^ The day of initiation of hormonal stimulation. ^3^ The day of the FTAI (at the completion of hormonal stimulation). ^4^ The day of the post-FTAI. A,B Values with different superscript letters indicate significant differences between mAFC ipsilateral and contralateral to the DF ovary at *p*-value < 0.05. a,b Values with different superscript letters indicate significant differences between mAFC on the left ovary and the right ovary in mAFC ipsilateral to the DF ovary at *p*-value < 0.05. AFC, antral follicle count; mAFC, main number of antral follicle populations; DF, dominant follicle; FTAI, fixed-time artificial insemination.

**Table 4:** Frequency of mAFC and DF on the left ovary and the right ovary and impact on mean number and diameter of AFC (mean ± SEM), ovarian response, and pregnancy outcomes in in vivo *Bos indicus* cow model with bilateral mAFC submitted to hormonal stimulation.

Items	Bilateral mAFC (*n* = 30) ^1^		*p*-Value
	mAFC and DF on the Left Ovary	mAFC and DF on the Right Ovary	
Total beef cows (*n*)	15	15	-
On day 0 ^2^			
Frequency relationship (%, *n*/*n*)	50.0 (15/30)	50.0 (15/30)	1.000
Mean number of AFC from left to right ovaries (*n*)	4.1 ± 0.44	5.2 ± 0.80	0.267
Mean diameter of AFC from left to right ovaries (mm)	4.0 ± 0.27	4.4 ± 0.32	0.326
On day 9 ^3^			
Beef cows DF > 8.5 mm in diameter (%, *n*/*n*)	53.3 (8/15)	73.3 (11/15)	0.264
Mean diameter of DF (mm)	9.4 ± 1.02	11.4 ± 0.63	0.116
On day 32 ^4^			
Pregnancy rate (%, *n*/*n*)	26.7 (4/15)	33.3 (5/15)	0.695

^1^ Bilateral mAFC means that the main number of antral follicle populations appears in equivalent numbers on both sides of the ovary. ^2^ The day of initiation of hormonal stimulation. ^3^ The day of the FTAI (at the completion of hormonal stimulation). ^4^ The day of the post-FTAI. AFC, antral follicle count; mAFC, main number of antral follicle populations; FTAI, fixed-time artificial insemination.

**Table 5:** Frequency of ovarian interrelationships and the impacts of ovarian interrelationships (unilateral and bilateral mAFC) on mean number and diameter of AFC (mean ± SEM), ovarian response, and pregnancy outcomes in primiparous cows submitted to hormonal stimulation.

Items	Ovarian Interrelationships (*n* = 29)		*p*-Value
	Unilateral mAFC ^1^	Bilateral mAFC ^2^	
Primiparous beef cows (*n*)	20	9	-
On day 0 ^3^			
Frequency relationship (%, *n*/*n*)	69.0 (20/29)	31.0 (9/29)	0.004
Mean number of AFC from left to right ovaries (*n*)	5.8 ± 0.55	4.2 ± 0.49	0.059
Mean diameter of AFC from left to right ovaries (mm)	4.2 ± 0.20	4.3 ± 0.41	0.867
On day 9 ^4^			
Primiparous cows DF > 8.5 mm in diameter (%, *n*/*n*)	75.0 (15/20)	55.6 (5/9)	0.304
Mean diameter of DF (mm)	10.8 ± 0.89	9.2 ± 1.14	0.305
On day 32 ^5^			
Pregnancy rate (%, *n*/*n*)	50.0 (10/20)	33.3 (3/9)	0.412

^1^ Unilateral mAFC means that the main number of antral follicle populations appears on only one side of the ovary. ^2^ Bilateral mAFC means that the main number of antral follicle populations appears in equivalent numbers on both sides of the ovary. ^3^ The day of initiation of hormonal stimulation. ^4^ The day of the FTAI (at the completion of hormonal stimulation). ^5^ The day of the post-FTAI. AFC, antral follicle count; mAFC, main number of antral follicle populations; FTAI, fixed-time artificial insemination.

**Table 6:** Frequency of unilateral mAFC relationships (mAFC ipsilateral and contralateral to the DF ovary) and impact on mean number and diameter of AFC (mean ± SEM), ovarian response, and pregnancy outcomes in primiparous cows submitted to the hormonal stimulation.

Items	Unilateral mAFC (*n* = 20) ^1^					
	mAFC Ipsilateral to the DF Ovary			mAFC Contralateral to the DF Ovary		
	mAFC on the Left Ovary	mAFC on the Right Ovary	Total	mAFC on the Left Ovary	mAFC on the Right Ovary	Total
Primiparous beef cows (*n*)	4	9	13	5	2	7
On day 0 ^2^						
Frequency relationship (%, *n*/*n*)	30.8 (4/13)	69.2 (9/13)	65.0 (13/20)	71.4 (5/7)	28.6 (2/7)	35.0 (7/20)
Mean number of AFC from left to right ovaries (*n*)	6.8 ± 0.89	6.0 ± 0.93	6.2 ± 0.71	5.0 ± 0.80	4.5 ± 1.77	4.9 ± 0.77
Mean diameter of AFC from left to right ovaries (mm)	4.1 ± 0.38	4.4 ± 0.30	4.3 ± 0.23	4.2 ± 0.36	4.0 ± 0.82	4.1 ± 0.36
On day 9 ^3^						
Primiparous cows DF > 8.5 mm in diameter (%, *n*/*n*)	30.8 (4/13)	53.8 (7/13)	55.0 (11/20) ^A^	42.9 (3/7)	14.3 (1/7)	20.0 (4/20) ^B^
Mean diameter of DF (mm)	12.6 ± 1.31	10.5 ± 1.19	10.5 ± 1.17	10.2 ± 2.31	10.0 ± 2.63	11.3 ± 1.33
On day 32 ^4^						
Pregnancy rate (%, *n*/*n*)	23.1 (3/13)	30.8 (4/13)	35.0 (7/20)	28.6 (2/7)	14.3 (1/7)	15.0 (3/20)

^1^ Unilateral mAFC means that the main number of antral follicle populations appears on only one side of the ovary. ^2^ The day of initiation of hormonal stimulation. ^3^ The day of the FTAI (at the completion of hormonal stimulation). ^4^ The day of the post-FTAI. A,B Values with different superscript letters indicate significant differences between mAFC ipsilateral and contralateral to the DF ovary at *p*-value < 0.05. AFC, antral follicle count; mAFC, main number of antral follicle populations; DF, dominant follicle; FTAI, fixed-time artificial insemination.

**Table 7:** Frequency of mAFC and DF on the left ovary and the right ovary and impact on mean number and diameter of AFC (mean ± SEM), ovarian response, and pregnancy outcomes in primiparous cows with bilateral mAFC submitted to hormonal stimulation.

Items	Bilateral mAFC (*n* = 9) ^1^		*p*-Value
	mAFC and DF on the Left Ovary	mAFC and DF on the Right Ovary	
Primiparous beef cows (*n*)	7	2	-
On day 0 ^2^			
Frequency relationship (%, *n*/*n*)	77.8 (7/9)	22.2 (2/9)	0.022
Mean number of AFC from left to right ovaries (*n*)	3.7 ± 0.48	6.0 ± 0.00	0.005
Mean diameter of AFC from left to right ovaries (mm)	4.4 ± 0.50	4.0 ± 0.55	0.708
On day 9 ^3^			
Primiparous cows DF > 8.5 mm in diameter (%, *n*/*n*)	57.1 (4/7)	50.0 (1/2)	0.866
Mean diameter of DF (mm)	9.0 ± 1.39	9.9 ± 1.51	0.747
On day 32 ^4^			
Pregnancy rate (%, *n*/*n*)	28.6 (2/7)	50.0 (1/2)	0.593

^1^ Bilateral mAFC means that the main number of antral follicle populations appears in equivalent numbers on both sides of the ovary. ^2^ The day of initiation of hormonal stimulation. ^3^ The day of the FTAI (at the completion of hormonal stimulation). ^4^ The day of the post-FTAI. AFC, antral follicle count; mAFC, main number of antral follicle populations; FTAI, fixed-time artificial insemination.

**Table 8:** Frequency of ovarian interrelationships and the impacts of ovarian interrelationships (unilateral and bilateral mAFC) on mean number and diameter of AFC (mean ± SEM), ovarian response, and pregnancy outcomes in multiparous cows submitted to hormonal stimulation.

Items	Ovarian Interrelationships (*n* = 75)		*p*-Value
	Unilateral mAFC ^1^	Bilateral mAFC ^2^	
Multiparous beef cows (*n*)	54	21	-
On day 0 ^3^			
Frequency relationship (%, *n*/*n*)	72.0 (54/75)	28.0 (21/75)	0.001
Mean number of AFC from left to right ovaries (*n*)	4.2 ± 0.29	4.9 ± 0.63	0.389
Mean diameter of AFC from left to right ovaries (mm)	4.6 ± 0.20	3.8 ± 0.18	0.190
On day 9 ^4^			
Multiparous cows DF > 8.5 mm in diameter (%, *n*/*n*)	85.2 (46/54)	66.7 (14/21)	0.074
Mean diameter of DF (mm)	11.1 ± 0.43	9.7 ± 0.90	0.825
On day 32 ^5^			
Pregnancy rate (%, *n*/*n*)	29.6 (16/54)	28.6 (6/21)	0.928

^1^ Unilateral mAFC means that the main number of antral follicle populations appears on only one side of the ovary. ^2^ Bilateral mAFC means that the main number of antral follicle populations appears in equivalent numbers on both sides of the ovary. ^3^ The day of initiation of hormonal stimulation. ^4^ The day of the FTAI (at the completion of hormonal stimulation). ^5^ The day of the post-FTAI. AFC, antral follicle count; mAFC, main number of antral follicle populations; FTAI, fixed-time artificial insemination.

**Table 9:** Frequency of unilateral mAFC relationships (mAFC ipsilateral and contralateral to the DF ovary) and impact on mean number and diameter of AFC (mean ± SEM), ovarian response, and pregnancy outcomes in multiparous cows submitted to the hormonal stimulation.

Items	Unilateral mAFC (*n* = 54) ^1^					
	mAFC Ipsilateral to the DF Ovary			mAFC Contralateral to the DF Ovary		
	mAFC on the Left Ovary	mAFC on the Right Ovary	Total	mAFC on the Left Ovary	mAFC on the Right Ovary	Total
Multiparous beef cows (*n*)	9	22	31	9	14	23
On day 0 ^2^						
Frequency relationship (%, *n*/*n*)	29.0 (9/31) ^b^	71.0 (22/31) ^a^	57.4 (31/54)	39.1 (9/23)	60.9 (14/23)	42.6 (23/54)
Mean number of AFC from left to right ovaries (*n*)	3.7 ± 0.52	4.1 ± 0.41	4.0 ± 0.33	4.3 ± 0.52	4.8 ± 0.81	4.7 ± 0.76
Mean diameter of AFC from left to right ovaries (mm)	4.4 ± 0.39	4.3 ± 0.34	4.3 ± 0.27	4.5 ± 0.26	5.2 ± 0.45	4.9 ± 0.30
On day 9 ^3^						
Multiparous cows DF > 8.5 mm in diameter (%, *n*/*n*)	25.8 (8/31) ^b^	58.1 (18/31) ^a^	48.1 (26/54)	34.8 (8/23)	52.2 (12/23)	37.0 (20/54)
Mean diameter of DF (mm)	10.5 ± 0.56	11.2 ± 0.74	10.9 ± 0.58	11.7 ± 0.92	10.9 ± 0.94	11.4 ± 0.64
On day 32 ^4^						
Pregnancy rate (%, *n*/*n*)	3.2 (1/31) ^b^	22.6 (7/31) ^a^	14.8 (8/54)	8.7 (2/23)	26.1 (6/23)	14.8 (8/54)

^1^ Unilateral mAFC means that the main number of antral follicle populations appears on only one side of the ovary. ^2^ The day of initiation of hormonal stimulation. ^3^ The day of the FTAI (at the completion of hormonal stimulation). ^4^ The day of the post-FTAI. a,b Values with different superscript letters indicate significant differences between mAFC on the left ovary and the right ovary in mAFC ipsilateral to the DF ovary at *p*-value < 0.05. AFC, antral follicle count; mAFC, main number of antral follicle populations; DF, dominant follicle; FTAI, fixed-time artificial insemination.

**Table 10:** Frequency of mAFC and DF on the left ovary and the right ovary and impact on mean number and diameter of AFC (mean ± SEM), ovarian response, and pregnancy outcomes in multiparous cows with bilateral mAFC submitted to hormonal stimulation.

Items	Bilateral mAFC (*n* = 21) ^1^		*p*-Value
	mAFC and DF on the Left Ovary	mAFC and DF on the Right Ovary	
Multiparous beef cows (*n*)	8	13	-
On day 0 ^2^			
Frequency relationship (%, *n*/*n*)	38.1 (8/21)	61.9 (13/21)	0.127
Mean number of AFC from left to right ovaries (*n*)	4.5 ± 0.68	5.1 ± 0.91	0.636
Mean diameter of AFC from left to right ovaries (mm)	3.6 ± 0.16	4.5 ± 0.35	0.046
On day 9 ^3^			
Multiparous cows DF > 8.5 mm in diameter (%, *n*/*n*)	50.0 (4/8)	76.9 (10/13)	0.215
Mean diameter of DF (mm)	9.7 ± 1.46	11.6 ± 0.67	0.176
On day 32 ^4^			
Pregnancy rate (%, *n*/*n*)	25.0 (2/8)	30.8 (4/13)	0.782

^1^ Bilateral mAFC means that the main number of antral follicle populations appears in equivalent numbers on both sides of the ovary. ^2^ The day of initiation of hormonal stimulation. ^3^ The day of the FTAI (at the completion of hormonal stimulation). ^4^ The day of the post-FTAI. AFC, antral follicle count; mAFC, main number of antral follicle populations; FTAI, fixed-time artificial insemination.

## Data Availability

The data that support the findings of this study are available from the corresponding author upon reasonable request.

## Supplementary Materials

The following supporting information can be downloaded at: https://www.mdpi.com/article/10.3390/biology11040523/s1, Figure S1: Sonogram of beef cows bearing antral follicles (A) or dominant follicle (B) on their ovary; Figure S2: Sonogram of beef cows were defined as pregnant (A) and non-pregnant (B) statuses at 32 days post-FTAI.
