# Supplementary material for: Understanding the Ovarian Interrelationship with Low Antral Follicle Counts (AFC) in the In Vivo Bos indicus Cow Model: Unilateral and Bilateral Main AFC as Possible Biomarkers of Ovarian Response to Hormonal Synchronisation
Source: Biology (Basel). 2022 Mar 29;11(4):523. doi: 10.3390/biology11040523 (PMC9029639; doi:10.3390/biology11040523)
Supplement: Supplementary file 1 [file biology-11-00523-s001.zip › biology-1562039-supplementary.pdf]

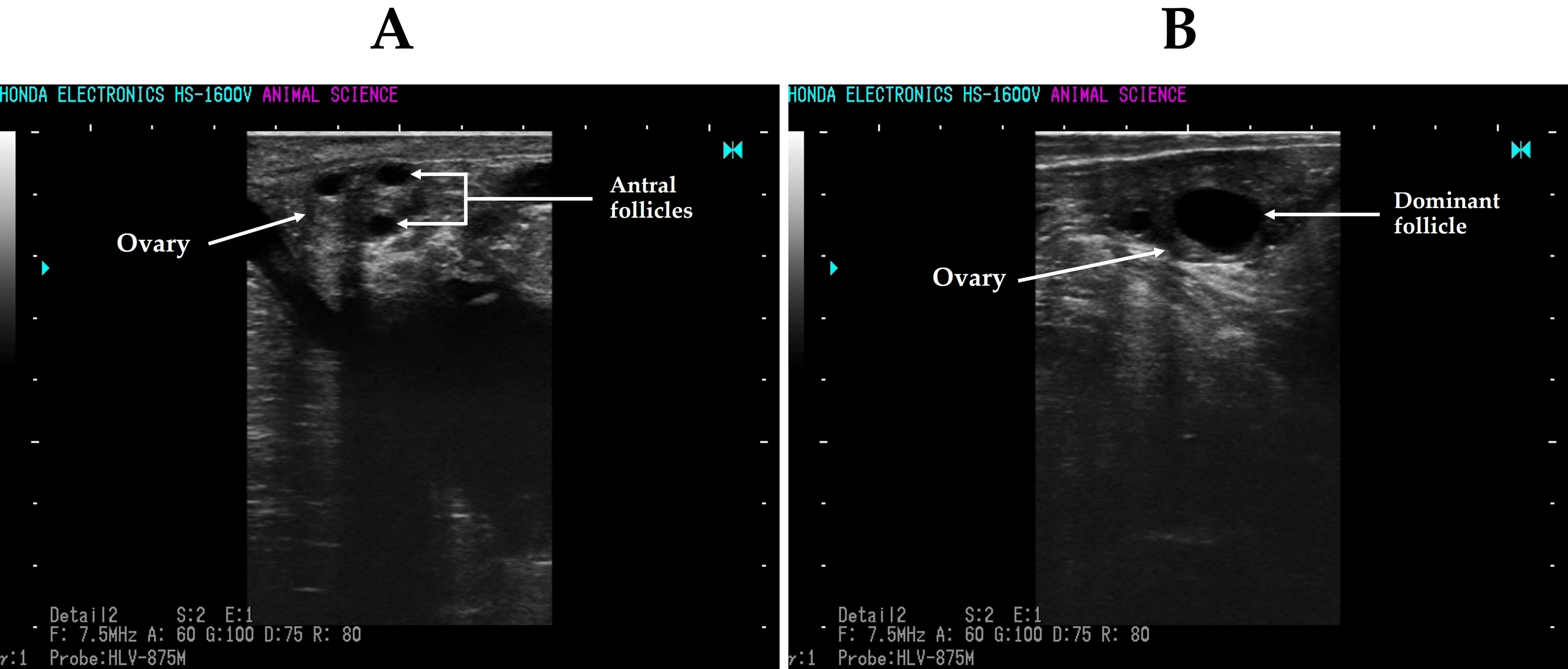
A
B
HONDA ELECTRONICS HS-1600V ANIMAL SCIENCE
Antral follicles
Ovary
Detail2 S:2 E:1
F: 7.5MHz A: 60 G:100 D:75 R: 80
Probe:HLV-875M
HONDA ELECTRONICS HS-1600V ANIMAL SCIENCE
Dominant follicle
Ovary
Detail2 S:2 E:1
F: 7.5MHz A: 60 G:100 D:75 R: 80
Probe:HLV-875M

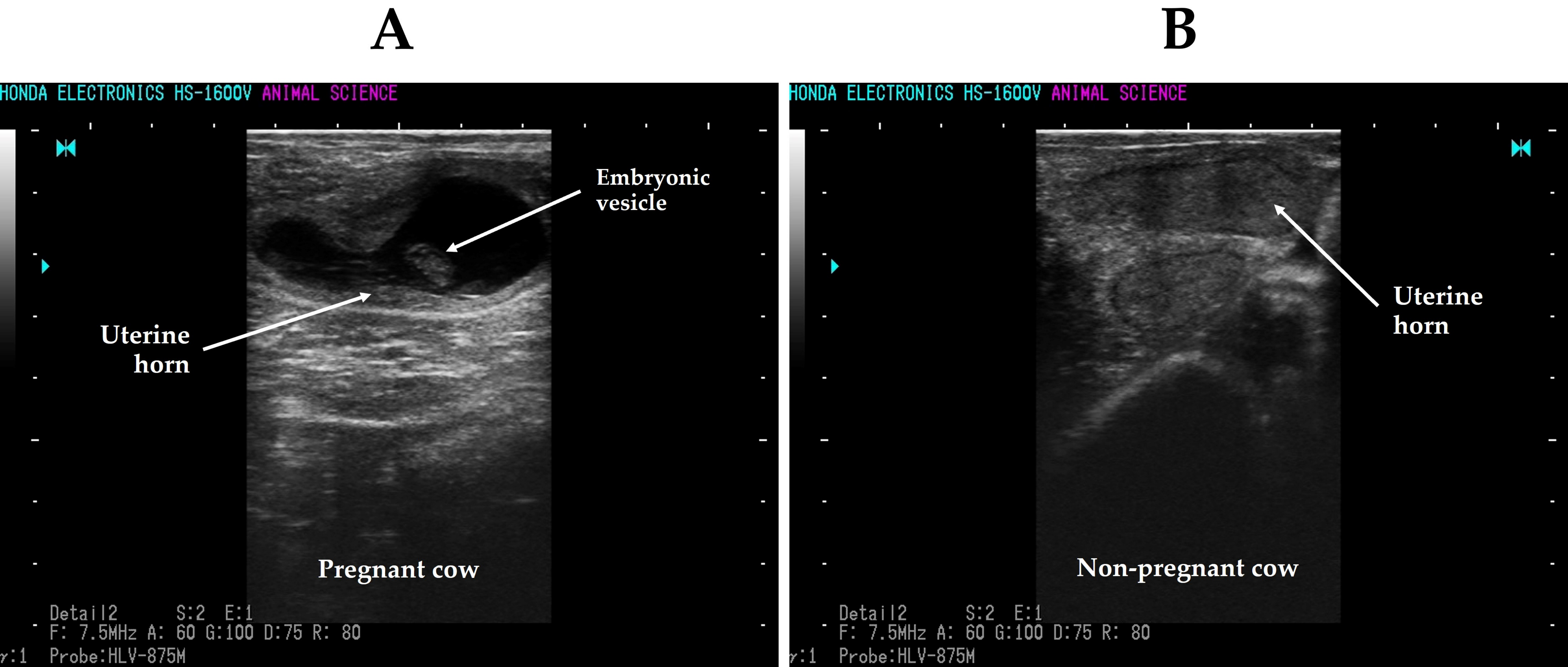
A
HONDA ELECTRONICS HS-1600V ANIMAL SCIENCE
Embryonic vesicle
Uterine horn
Pregnant cow
Detail2 S:2 E:1
F: 7.5MHz A: 60 G:100 D:75 R: 80
Probe:HLV-875M
B
HONDA ELECTRONICS HS-1600V ANIMAL SCIENCE
Uterine horn
Non-pregnant cow
Detail2 S:2 E:1
F: 7.5MHz A: 60 G:100 D:75 R: 80
Probe:HLV-875M
